# Clinical Diagnosis and Treatment in Patients with Schwannoma at a Single Institute

**DOI:** 10.3390/diagnostics16152299

**Published:** 2026-07-23

**Authors:** Tomoki Nakamura, Ryohei Adachi, Tomohito Hagi, Yumi Matsuyama, Hironori Date, Masahiro Hasegawa

**Affiliations:** Department of Orthopaedic Surgery, Graduate School of Medicine, Mie University, Tsu 514-8507, Japan

**Keywords:** schwannoma, neurological deficit, clinical outcome

## Abstract

**Background/Objectives**: The aim of this study was to review the clinical course of schwannoma in 176 patients and elucidate the clinical outcome in a patient who received surgical treatment and one who did not. **Methods**: Between August 2006 and December 2021, 176 patients with solitary and sporadic schwannomas were enrolled in our institution. There were 94 men and 82 women, with a mean age of 57 years. During follow-up, 76 patients received surgical treatment, and the remaining 100 did not. **Results**: The mean tumor size at initial presentation was 30 mm. The major tumor locations were the thigh, lower leg, upper arm, and forearm. The major nerves affected by the schwannoma were the intramuscular branch, sciatic nerve, and ulnar nerve. At least one symptom in addition to a palpable mass was observed in 133 patients (76%). The variables that indicated surgical treatment were tumor size at initial presentation, age, and any other existing symptoms. After surgery, pain-related symptoms including pain, irradiated pain and tenderness were resolved completely in 42 of 44 patients (95%) who had displayed them preoperatively. Neurological deficit was observed in 26 patients (34%). Among the 26 patients with neurological deficit, neurological sensory deficit was observed in 23 patients, and the remaining three had sensory and motor deficits. Multivariate analysis showed that patients with major nerve schwannoma, those with paresthesia preoperatively, and those who developed preoperative symptoms and had undergone surgery had a higher risk of the development of postoperative neurological deficit. **Conclusions:** Our findings should be considered while planning surgery and obtaining informed consent.

## 1. Introduction

Schwannomas are common benign soft tissue tumors and account for 5% of all soft tissue tumors, occurring between the ages of 20 and 60 years, and display no significant sex bias [1,2,3,4,5]. Schwannomas are also the most frequent type of peripheral nerve tumors [2,5]. Schwannomas are usually isolated, solitary, and well-encapsulated lesions, but they may be associated with schwannomatosis and neurofibromatosis type 2 [6,7,8]. Physical examination is the first step in confirming the diagnosis of schwannoma. A palpable mass and Tinel’s sign are apparently the two most common and well-cited clinical findings of peripheral nerve tumors [2,5,8]. In addition to Tinel’s sign, the clinical symptoms of schwannoma may include local pain, muscle weakness, and paresthesia. On examination, around 20% of patients with schwannoma display sensory disturbance, in the area of the affected nerve [9]. The occurrence of motor deficit is rare. Magnetic resonance imaging (MRI) is considered the most useful technique for diagnosing schwannoma. MRI conducted in patients with schwannoma shows homogeneous low signal intensity on T1-weighted images and high signal intensity on T2-weighted images. Notably, the presence of a target sign is known as one of the characteristic imaging findings in schwannomas [8]. The target sign has been described as a central/peripheral biphasic pattern detected on MRI radiographs and has been used to diagnose schwannomas. A split fat sign is also detected, which is characterized by a rim of fat around the tumor [8]. The sensitivity of preoperative MRI to correctly identify upper limb schwannomas ranges between 85 and 91% [2,10]. Schwannomas are slow-growing tumors and often present as asymptomatic masses or incidental findings on imaging surveys such as MRI and computed tomography (CT). In symptomatic cases, surgery may be considered. Encapsulation is a major characteristic of schwannomas and, therefore, theoretically allows for their enucleation without nerve damage [11,12,13,14]. However, some schwannomas cannot be easily enucleated because of the risk of causing an iatrogenic nerve injury. Even if complete enucleation was carried out via a microscopic procedure, neurological complications may develop. Sensory neurological deficits were reported to range from 3 to 67%, while motor neurological deficits were found to be rare [11,12,13,14,15,16,17]. Therefore, for patients with asymptomatic and minor symptomatic symptoms, there are no recommendations about surgical treatment. The aim of this study was to review the clinical course of the disease in 176 patients with solitary and sporadic schwannoma and elucidate the clinical outcome in a patient who received surgical treatment and one who did not.

## 2. Materials and Methods

**Figure 1:**
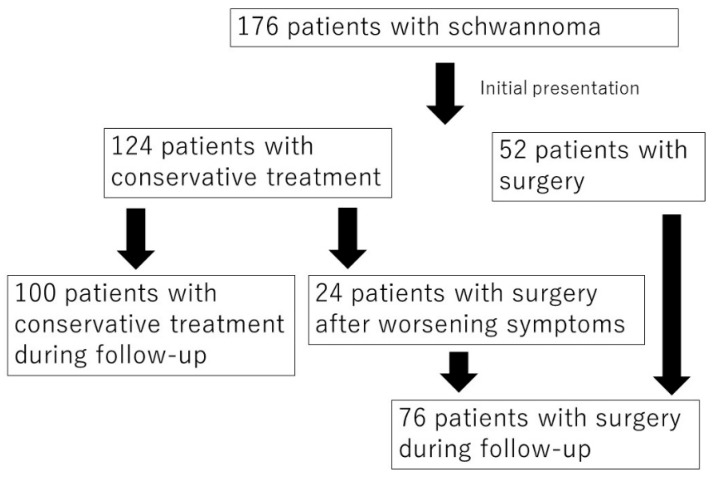
A schema for the clinical course of the disease in 176 patients.

Between August 2006 and December 2021, 176 patients with solitary and sporadic schwannomas were enrolled in our institution. There were 94 men and 82 women, with a mean age of 57 years (range, 14–89). We excluded deep axial schwannomas such as nerve root, intradural extramedullary, retroperitoneal, intraabdominal, and pelvic cavity schwannomas. We also excluded multiple schwannomas and neurofibromatosis type 2. MRI was carried out in all patients. Of the 176 patients included in this study, a pathological diagnosis was confirmed in 87, including 11 patients who underwent biopsy alone and 76 patients who received surgical treatment. These 76 patients comprised two groups: one containing the patients who underwent surgery just after initial presentation (*n* = 52) and another where patients underwent surgery after their symptoms had worsened (*n* = 26). A biopsy was carried out in 21 patients. Generally, a biopsy was conducted in patients with tumors > 5 cm at our institution. During follow-up, 100 patients did not receive surgical treatment (Figure 1). A clinical diagnosis that included physical examination and MRI findings was confirmed in the remaining 89 patients. MRI findings detected the target sign in 79 patients, while its absence was noted in 10 patients. Patients were operated upon via a microsurgical approach with magnification loupes under general or loco-regional anesthesia. Wherever possible, we applied an air tourniquet to maintain the visualization of the surgical field. The tumor was exposed along with the affected nerve. Some nerve fascicles were observed to be running on the surface of the tumor. The dissection was performed carefully on the epineurial area devoid of fascicles. The epineural capsule was carefully peeled away to enucleate the tumor. If an iatrogenic nerve injury was predicted, we did not carry out a complete tumor enucleation. All 76 patients were treated via the enucleation technique. One patient with a schwannoma located in the brachial plexus received additional en bloc surgical resection due to growth after incomplete enucleation. We evaluated postoperative neurological deficit. We defined neurological deficit as the existence of neurological symptoms at the final follow-up time with a minimum follow-up duration of 3 months. Neurological deficit included sensory disturbance and motor deficit. Sensory disturbance included hypoesthesia and paresthesia.

### Statistical Analysis

Clinicopathological factors were evaluated using the Mann–Whitney U test (quantitative data) and Fisher’s exact test (qualitative data). Spearman’s rank correlation coefficient test was employed to analyze the relationship between tumor growth rate, age, and tumor size. Logistic multivariate analysis using a forward–backward stepwise method was used for predicting the development of postoperative neurological deficit. Although age and tumor size were quantitative data, we created 2 groups according to the median age and tumor size in patients who received surgery. All statistical analyses were conducted using the EZR graphical user interface (Saitama Medical Center, Jichi Medical University, Saitama, Japan) for R software version 1.70 (R Foundation for Statistical Computing, Vienna, Austria), a modified version of R Commander with functions commonly used in biostatistics. *p* values < 0.05 were considered statistically significant.

## 3. Results

### 3.1. Patient Characteristics

There were 176 patients diagnosed with schwannoma. The mean tumor size at initial presentation was 30 mm (range, 7–118). Tumor locations were as follows: thigh, 38 cases; leg, 25 cases; upper arm, 20 cases; forearm, 19 cases; knee, 18 cases; neck, 10 cases; hand and finger, 9 cases; chest wall, 8 cases; foot and ankle, 8 cases; axilla, 5 cases; elbow, 5 cases; and other locations, 11. The affected nerves were the intramuscular branch (*n* = 35), sciatic nerve (*n* = 28), ulnar nerve (*n* = 20), cutaneous nerve (*n* = 19), brachial plexus (*n* = 15), tibial nerve (*n* = 14), median nerve (*n* = 12), peroneal nerve (*n* = 9), radial nerve (*n* = 9), digital nerve (*n* = 5), femoral nerve (*n* = 4), sural nerve (*n* = 3), obturator nerve (*n* = 2), and intracostal nerve (*n* = 1) (Table 1).

Regarding patient symptoms, local pain was observed in 23 patients (13%), tenderness in 61 (35%), irradiated pain in 45 (26%), and paresthesia (numbness and tingling sensations) in the area of the affected nerve in 35 (20%). At least one symptom was observed in 133 patients (76%). Tinel’s sign was found in 80 (45%) patients. On MRI radiographs, the target sign was observed in 133 patients (76%) (Table 2). A biopsy was conducted in 21 patients (12%) and for large-sized tumors. The median tumor size in patients who underwent the biopsy procedure was 53.5 mm, compared to 25 mm in patients who did not undergo a biopsy (*p* < 0.0001, Mann–Whitney U test). A biopsy was frequently carried out in patients not displaying the target sign on MRI radiographs. Among 43 patients not displaying the target sign on MRI radiographs, 14 patients (33%) underwent a biopsy procedure compared with 10 out of 133 patients (8%) who presented with a palpable mass as well as the target sign (*p* = 0.000128, Fisher’s exact test). Among 21 patients who underwent a biopsy, one patient developed paresthesia after biopsy.

Of the 176 patients in this study, 76 (43%) underwent surgical treatment, and the remaining 100 did not. Of the 100 patients who did not undergo surgery, conservative treatment was selected for 21 due to a high risk of postoperative neurological symptoms if the tumor was enucleated. Severe symptoms were not observed, and surgical treatment was not initiated in the remaining 79 patients. The variables that indicated surgical treatment were tumor size (*p* = 0.0432, Mann–Whitney U test), age (*p* = 0.00351, Mann–Whitney U test), and any other existing symptoms (*p* = 0.0125, Fisher’s exact test). There was no relationship between surgical treatment and individual symptoms such as local pain and paresthesia, although patients with tenderness did undergo surgery (*p* = 0.08, Mann–Whitney U test) (Table 3).

#### 3.1.1. Clinical Outcomes After Surgery

Of the 176 patients in this study, 76 (43%) underwent surgical treatment. The median duration between initial presentation and surgery was 104 days. Of the 76 patients who underwent surgery, 52 (68%) decided in favor of surgical treatment at the time of initial presentation at our institution. The remaining 24 patients (32%) underwent surgery when their symptoms began to worsen. The median duration between initial presentation and surgery was 22.7 months for patients who developed symptoms preoperatively and underwent surgery compared with 2.3 months for those who elected surgery at the time of initial presentation (*p* < 0.0001, Mann–Whitney U test). Enucleation was performed in all patients at the time of initial surgery. One patient underwent additional en bloc resection due to incomplete enucleation and mass growth postoperatively. The symptoms indicating surgery were as follows: tenderness (*n* = 20), irradiated pain (*n* = 9), local pain (*n* = 7), paresthesia (numbness and tingling sensations in the area of the affected nerve) (*n* = 7), irradiated pain and tenderness (*n* = 6), irradiated pain and paresthesia (*n* = 5), tenderness and paresthesia (*n* = 4), local pain and tenderness (*n* = 3), local pain and paresthesia (*n* = 1), and tenderness, irradiated pain and paresthesia (*n* = 1). The remaining 14 patients decided in favor of surgery to resect the tumor.

The mean and median durations (months) between the date of surgery and final follow-up were 26.4 and 14.2 months, respectively. Pain-related symptoms including pain, irradiated pain and tenderness were resolved completely in 54 of 56 patients (96%) who displayed them preoperatively. Pain improved in the remaining two patients who had no medication. Neurological deficit was observed in 26 patients (34%) postoperatively. Of the 26 patients with postoperative neurological deficit, neurological sensory disturbance was observed in 23, and the remaining three had sensory disturbance and motor deficit. Sensory disturbance included hypoesthesia (*n* = 3) and paresthesia (*n* = 23). An improvement in paresthesia was observed in four of 15 patients (27%) who had preoperative paresthesia. Of these four, two patients developed hypoesthesia instead of paresthesia. Paresthesia persisted in nine of 15 patients (60%) postoperatively. Paresthesia was completely resolved in two of 15 patients (13%) who had displayed it preoperatively. Of the 61 patients who did not display sensory disturbance preoperatively, 13 (21%) developed it, and one patient (1%) developed paresthesia and hypoesthesia postoperatively, respectively (Table 4).

We analyzed the relationship between postoperative neurological deficit and patient background. We defined the major motor nerves including motor bundles, the loss of which affects the performance of daily activities, including the following: brachial plexus, median nerve, ulnar nerve, radial nerve, musculocutaneous nerve, posterior interosseous nerve, obturator nerve, sciatic nerve, femoral nerve, tibial nerve, common peroneal nerve, and deep peroneal nerve based on the findings of Hirai et al. [11]. Patients with major nerve schwannoma frequently developed postoperative neurological deficit (*p* < 0.0001, Fisher’s exact test). Of the 44 patients with major motor nerve schwannoma, 23 (52%) developed postoperative neurological deficit, while 2 of 32 (6%) patients developed other nerve schwannomas. Patients with paresthesia as a preoperative symptom and/or major motor nerve schwannoma frequently developed postoperative neurological deficit. Further, postoperative neurological deficit was frequently observed in patients who developed preoperative symptoms and had undergone surgery, compared with those who elected surgery at the time of initial presentation. Of the 52 patients who underwent surgery after initial presentation, 12 (23%) developed postoperative neurological deficit, while 14 of 24 (58%) patients developed other nerve schwannomas (Table 5). Multivariate analysis showed that patients with major nerve schwannoma, those with paresthesia preoperatively, and those who developed preoperative symptoms and had undergone surgery had a higher risk of the development of postoperative neurological deficit (Table 6).

#### 3.1.2. The Case Presentation of a Patient Who Underwent Surgery

The patient was a 54-year-old man. His complaint was paresthesia at the right extremity. Paresthesia was observed at the right shoulder and the lateral side of the upper arm. Tinel’s sign was not observed. MRI showed low signal intensity on T1-weighted radiographs and heterogenous to high intensity on T2-weighted radiographs. After the administration of gadolinium-containing contrast medium, the tumor revealed heterogeneous enhancement. The size of the tumor was 84 by 64 by 38 mm, and it was located in the brachial plexus. Due to severe paresthesia, surgical treatment was requested. Although the presence of a schwannoma was suspected, we performed intraoperative frozen section diagnosis due to the large size of the tumor. The intraoperative frozen section findings revealed a schwannoma, and enucleation was conducted. However, complete enucleation was not carried out due to the high risk of nerve injury. Two years postoperatively, paresthesia had persisted but had improved. The extension of the right shoulder was impossible, and atrophy of the deltoid muscle was observed. There was no restriction of the activities of daily living. The residual tumor was observed on MRI radiographs.

#### 3.1.3. Clinical Course in Patients Without Surgery

Of the 176 patients included in this study, conservative treatment was initiated in 124. Biopsy was performed in 16 patients (13%). Of the 124 patients who underwent a biopsy, surgical treatment was carried out in 24 during follow-up due to increasing symptoms. Of the 100 patients who did not undergo surgery, follow-up MRI was performed in 66 at our institution. The remaining 34 patients were referred to previously visited hospitals. The mean and median sizes of the tumor at initial presentation were 31 mm and 25 mm (range, 8–118), respectively. The mean and median sizes of the tumor at final presentation were 34 mm and 27 mm (range, 8–120), respectively. The mean and median follow-up days between initial and final presentation at our institution were 77.3 months and 45.5 months, respectively.

We employed MRI to measure the tumor size in 66 patients who were followed up. The ratio between the maximum diameter at initial MRI to that at the latest MRI varied from 0.7 to 2.0. The growth rate was not associated with tumor size and age at initial presentation. There were no cases of malignant transformation during the follow-up period.

#### 3.1.4. The Case Presentation of a Patient Who Did Not Undergo Surgery

**Figure 2:**
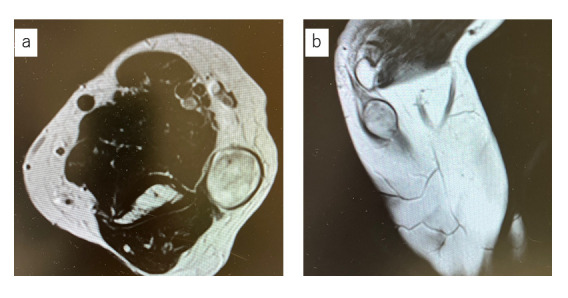
T2-weighted radiographs acquired by magnetic resonance imaging (MRI). (**a**) The target sign was observed in the axial view at the left elbow. (**b**) The target sign and split fat sign were observed in the sagittal view at the left elbow.

The patient was a 68-year-old woman. Her complaint was a mass at the left elbow and irradiated pain. Irradiated pain was observed at the ulnar nerve area, especially the ring and little fingers. Tinel’s sign was observed. MRI findings showed low signal intensity on T1-weighted radiographs and low to intermediate signal intensity with a peripheral ring of high signal intensity (target sign) on T2-weighted radiographs in the axial view. The split fat sign was observed in the sagittal view (Figure 2). After the administration of gadolinium-containing contrast medium, the tumor revealed heterogeneous enhancement. The size of the tumor was 20 × 18 × 18 mm, and it was located on the ulnar nerve at the elbow. Due to the risk of postoperative neurological deficit, conservative treatment was carried out. Pregabalin was administered at a dosage dependent on the degree of pain. After 6.5 years, the size of the tumor had progressed to 25 × 21 × 17 mm as revealed by MRI carried out at the last presentation.

## 4. Discussion

In this study, we analyzed 176 patients with schwannomas. Of the 176 patients included in this study, 76 (43%) underwent surgical treatment. After surgery, pain related symptoms were completely resolved for 42 of 44 patients (95%) who had them preoperatively. Neurological deficit was observed in 26 patients (34%).

For the diagnosis of schwannoma, a palpable mass with Tinel’s sign is apparently the most common and most cited clinical finding of peripheral nerve tumors. Preoperatively, approximately 20% of patients with schwannoma showed sensory disturbance in the area of the affected nerve [2,5,8,9]. In this study, Tinel’s sign was found in 80 (45%) patients and paresthesia in the area of the affected nerve in 35 (20%) patients, although there was no patient with motor deficit preoperatively. These findings are consistent with previous studies. MRI is considered the most useful technique for diagnosing schwannoma due to recent advances in this methodology [8,10,18,19,20,21]. Notably, the target sign is known as one of the characteristic imaging findings in schwannomas [8,10]. Shimose et al. reported the presence of the target sign in 50% of patients with schwannoma. They also reported no significant differences in target sign occurrence on MRI radiographs between major nerve schwannomas and intramuscular schwannomas [20]. In this study, the target sign was observed in 133 patients (76%) on MRI findings. Therefore, the radiological diagnosis of a peripheral schwannoma may prove straightforward for most patients with typical findings in addition to physical examination. However, there are some patients for whom differentiating benign from soft tissue sarcoma can be challenging [22,23]. The indication of biopsy is debated in peripheral nerve tumors. Although rare, complications from needle or open biopsy have been described [24,25]. Biopsy may lead to peritumoral fibrosis and increase difficulty in complete enucleation. Perez-Roman et al. analyzed 151 patients with peripheral nerve tumors, including 88 schwannomas. Nineteen patients with schwannoma underwent biopsy, and nine patients (47%) showed increased neurological deficit from the biopsy alone per comprehensive history and physical exam [23]. In this study, biopsy was performed in 21 patients (12%), and one patient (5%) developed paresthesia. Biopsy was frequently performed in patients with a large-sized tumor or no target sign detected in MRI findings. When typical clinical findings were absent such as atypical MRI findings or the presence of a large tumor, a biopsy may be considered, although the evaluation of the possible risk of nerve injury should be carried out.

The indication for surgical treatment for schwannomas is still controversial due to the risk of iatrogenic nerve injury [11,12,13,14]. In this study, 43 of 76 patients (57%) displayed symptoms that indicated surgical treatment. Among these 76 patients, 24 (32%) underwent surgery when their symptoms were worsening, while 21 of 100 patients (21%) who did not undergo surgery chose conservative treatment due to the risk of nerve injury and postoperative neurological deficit. In our institution, carrying out surgery is decided after a careful discussion of the risks and benefits of surgery with the patient. We primarily carry out extracapsular resection, removing the entire mass after opening the epineurium and identifying the fascicle around the schwannoma, if possible. If an iatrogenic nerve injury is predicted, we do not perform complete tumor enucleation and do piece-by-piece resection. The rate of the complete removal of the tumor in this study was not calculated because of the lack of these data in some patients, although one patient with a schwannoma at the brachial plexus received additional en bloc surgical resection due to mass growth after incomplete enucleation.

The incidence of neurological complaints after surgery for schwannomas has been reported to vary from 3 to 67%, which is consistent with the present study (33%) [11,12,13,14,15,16,17]. We found that patients with paresthesia as a preoperative symptom and/or main trunk schwannoma frequently developed postoperative neurological deficit. Raj et al. reported clinical outcomes in 71 patients with limb schwannomas [14]. After surgery, neurological deficit was found in 25 patients. Neurological deficit was sensory in 20 patients and motor in nine patients. They found that fascicular resection was associated with the risk of postoperative deficit. Fujibuchi et al. analyzed 95 patients with schwannomas in the extremities and the trunk [13]. Postoperative neurological complications were observed in 18.4% of patients. They found that preoperative sensory disturbance and a tumor located in the proximal part of the limbs were independent risk factors for neurological complications postoperatively. Hirai et al. reported clinical outcomes in 139 patients with 141 schwannomas arising in major nerves [11]. Postoperative complications occurred in 49 lesions (34.8%), including 42 with sensory disturbance and 8 with motor weakness. They concluded that major motor nerve involvement was an independent predictive factor for postoperative complications. In the present study, patients with paresthesia as a preoperative symptom and/or major motor nerve schwannoma frequently developed postoperative neurological deficit. Granlund et al. reported that only 3% (3 of 99) of patients developed nerve palsies, although they did not include hypoesthesia. They speculated that the differences in the rate of postoperative neurological deficit were due to the vast variation in follow-up periods and differences in the definition of neurological deficits [17]. Age and the size of the schwannoma were reported to be risk factors for postoperative neurologic deficit [26]. However, in our present study, these features were not identified as risk factors. Interestingly, postoperative neurological deficit was frequently detected in patients who developed preoperative symptoms and underwent surgery, compared with those who elected surgery at the time of initial presentation. The worse preoperative symptoms during follow-up may be related to the occurrence of postoperative neurological deficit. Further studies will be necessary to validate these findings.

Regarding the clinical course of postoperative neurological deficit, several studies reported that both sensory disturbance and motor deficits improved to some degree in 73–100% of patients [11,17]. They unanimously showed that most sensory disturbances caused by surgery improved subsequently to full recovery or mild disorders with no need for medication or nerve block. On the other hand, many preceding studies reported that postoperative muscle weakness was not fully resolved [11,17]. Similar results were obtained in our current study. In the present study, the recovery of muscle power was not acquired in all three patients with motor deficits postoperatively after the passage of 2 years.

There were few reports concerning the natural history of schwannoma. Sayed et al. analyzed the tumor growth rate in 44 patients and 47 schwannomas [5]. They reported that the relative annual growth rate ranged from −9% to 166% with a mean annual rate of 33.9%. They also showed no association between tumor growth rate, tumor size, and age at diagnosis. Although we only carried out MRI two times (initial presentation and final follow-up), we also found no association between tumor growth rate, tumor size, and age at diagnosis. In this study, there were no patients with a malignant transformation occurring during their follow-up duration. The malignant transformation of a peripheral schwannoma is considered to be quite rare [27], although the malignant transformation of vestibular schwannomas after radiosurgery has been reported [28,29,30].

There were some limitations in this study. The symptoms were dependent on the claim of the patient. The degrees of pain, irradiated pain, and paresthesia were not measurable objectively. The follow-up duration was relatively short and varied among patients. Although we defined neurological deficit as the existence of neurological symptoms with a minimum follow-up duration of 3 months, follow-up duration varies substantially between patients, which may influence the reported incidence of postoperative complications. The residual neurological deficits may have further improved by employing longer follow-up durations. MRI was not routinely performed during follow-up. Therefore, the postoperative presence of residual tumor tissue was not confirmed in some patients on MRI radiographs. The intraoperative findings were not fully investigated, although fascicular resection may have been required in some patients. The retrospective nature of our study was another limitation. Treatment allocation was not random but based on physician and patient decision-making. Therefore, selection bias should be considered for interpreting the present results. Finally, because only 26 postoperative neurological deficit events were included in the multivariable analysis, the possibility of model overfitting should be considered.

## 5. Conclusions

We retrospectively analyzed 176 patients with schwannoma at a single institution. During follow-up, 43% of patients underwent surgery. After surgery, pain-related symptoms including pain, irradiated pain and tenderness were resolved completely in 95% of patients who had displayed them preoperatively. However, neurological deficit was observed in 26 patients (34%). Patients with paresthesia as a preoperative symptom and/or major motor nerve schwannoma frequently developed postoperative neurological deficit. Postoperative neurological deficit was also frequently observed in patients who had displayed this symptom preoperatively and underwent surgery, compared with those who elected to undergo surgery at the time of initial presentation. We conclude that these findings should be taken into consideration for planning surgery and obtaining informed consent.

## Figures and Tables

**Table 1:** Patient backgrounds.

Characteristics		*N*
Age	Mean	57 years
	Range	14–89 years
Sex	Man	94
	Woman	82
Nerve	Intramuscular	35
	Sciatic	28
	Ulnar	20
	Cutaneous	19
	Brachial plexus	15
	Tibial	14
	Median	12
	Peroneal	9
	Radial	9
	Digital	5
	Femoral	4
	Sural	3
	Obturator	2
	Intracostal	1

**Table 2:** Tumor characteristics.

Characteristics		Number
Size	Median	30 mm
	Range	7–118 mm
Target sign on MRI	Yes	133
	No	43
Symptom		
Local pain	Yes	23
	No	153
Tenderness	Yes	61
	No	115
Irradiated pain	Yes	45
	No	131
Paresthesia	Yes	35
	No	141
Tinel’s sign	Yes	80
	No	96
Biopsy	Yes	21
	No	155
Diagnosis	MRI with target sign	79
	MRI without target sign	10
	Biopsy	11
	Surgery	76

MRI, magnetic resonance imaging.

**Table 3:** Factors indicating surgical treatment.

Variables		Surgery		*p* Value
		Yes	No	
Sex	Man	44	50	0.36
	Woman	32	50	
Symptoms				
Any	Yes	61	62	0.0125
	No	15	38	
Pain	Yes	11	12	0.657
	No	65	88	
Irradiated pain	Yes	21	24	0.605
	No	55	76	
Tenderness	Yes	32	29	0.08
	No	44	71	
Numbness	Yes	17	18	0.568
	No	59	82	
Tumor size	Median	40 mm	34 mm	0.0432
Age	Median	52 years	62 years	0.00351

**Table 4:** Incidence of postoperative neurological deficit.

Preoperative Symptoms	*N*	Postoperative Neurological Deficit	
		Sensory (*n*)	Sensory and Motor (*n*)
Palpable mass alone	14	1	0
Pain	7	1	0
Irradiated pain	9	5	1
Tenderness	20	3	0
Paresthesia	7	4	2
Irradiated pain and paresthesia	5	3	0
Irradiated pain and tenderness	6	2	0
Pain and tenderness	3	0	0
Pain and paresthesia	1	0	0
Paresthesia and tenderness	4	2	0
Paresthesia, tenderness, and irradiated pain	1	1	0

**Table 5:** Risk factors for postoperative neurological deficit.

Variables		Neurological Deficit		*p* Value
		Yes	No	
Sex	Man	16	28	0.807
	Woman	10	22	
Symptoms				
Pain	Yes	1	10	0.0857
	No	25	41	
Irradiated pain	Yes	11	10	0.0579
	No	15	40	
Tenderness	Yes	9	24	0.332
	No	17	26	
Paresthesia	Yes	14	4	<0.0001
	No	12	46	
Target sign	Yes	19	32	0.455
	No	7	18	
Tumor size	Median	30	30	0.535
Age	Median	54	51	0.591
Major motor nerve	Yes	24	20	<0.0001
	No	2	30	
Surgery	Prompt	12	40	0.00407
	After worsening symptoms	14	10	

**Table 6:** Multivariate analysis for predicting postoperative neurological deficit.

Variables		Odds Ratio	95% CI	*p* Value
Age	>50 years	0.301	0.0503–1.8	0.189
	<50 years	1		
Sex	Male	0.658	0.141–3.07	0.594
	Female	1		
Symptom				
pain	Yes	0.162	0.00905–2.89	0.216
	No	1		
Irradiated pain	Yes	1.5	0.3–7.5	0.621
	No	1		
Tenderness	Yes	0.474	0.0913–2.46	0.375
	No	1		
Paresthesia	Yes	9.41	1.43–61.7	0.0195
	No	1		
Target sign	Yes	0.293	0.0472–1.82	0.188
	No	1		
Tumor size	>4 cm	3.07	0.511–18.4	0.221
	<4 cm			
Major motor nerve	Yes	11.2	1.63–77.4	0.014
	No	1		
Surgery	After worsening symptoms	13.2	1.9–91.6	0.00913
	Prompt	1		

## Data Availability

The original contributions presented in this study are included in the article. Further inquiries can be directed to the corresponding author.
